# Towards guiding principles in workflow design to facilitate collaborative projects involving massively parallel electrophysiological data

**DOI:** 10.1186/1471-2202-12-S1-P131

**Published:** 2011-07-18

**Authors:** Michael Denker, Andrew Davison, Markus Diesmann, Sonja Grün

**Affiliations:** 1 Laboratory for Statistical Neuroscience, RIKEN BSI, Wako-shi, 351-0198 Saitama, Japan; 2 Unité de Neurosciences, Information et Complexité (UNIC), CNRS UPR-3293, 91198 Gif sur Yvette, France; 3 Institute of Neuroscience and Medicine (INM-6), Research Center Jülich, 52428 Jülich, Germany

## 

The recent years have seen a rapid increase of interest in simultaneously analyzing the activity recorded from large numbers of channels in order to investigate the role of concerted neural activity in brain function. These efforts have led to advances in data analysis methods [1] that exploit the parallel properties of such data sets [2]. However, an often neglected aspect is that massively parallel data streams place new demands on handling their complexity during all stages of the project [3]: from the initial recording, throughout the analysis process, to the final publication. Three factors contribute these new demands: First, the sheer quantity of data complicates the organization of data sources, and the resulting automatization of analysis steps renders the validation of interim and final results difficult. Second, modern analysis methods often require intricate, multi-layered implementations, leading to sophisticated analysis toolchains [4]. Third, a growing number of projects needs to be carried out in teams, within a laboratory or in collaborative efforts, requiring transparent workflows that guarantee smooth interaction. Taken together, the increase in complexity calls for a reevaluation of the ad-hoc traditional approaches to such projects. Can we derive general guiding principles that may be adopted for designs of efficient workflows? How could these improve our confidence in handling the data by providing better cross-validation of findings, reliably managing provenance data, and enabling tighter collaborative research, while at the same time leaving the scientist with the flexibility required for creative research?

Although several projects are devoted to finding solutions for specific aspects of a workflow design (e.g., [5-7]), on a more general level there is lack of a thorough discussion on what goals are expected from a workflow, and which of these can be realistically addressed. Here, we summarize feedback received from experimenters and theoreticians that pinpoints the fundamental problems typically encountered in the analysis of high-dimensional electrophysiological data. Illustrated by examples from our own experience, we further show obstacles that prevent us from harmonizing workflows to common guidelines. For selected issues we draw parallels to other communities that are faced with similar problems (e.g., neuronal network modeling [8,9]; neuroimaging [10]). Lastly, we propose how existing concepts and software [9,11] could assist in practically implementing workflows that are tailored to the needs of a specific project, yet guarantee high standards by adhering to general guidelines of accepted best-practice.

## References

[B1] Brown EN Kass RE Mitra PP Multiple neural spike train data analysis: state-of-the-art and future challenges Nat Neurosci 2004 7 456 461 10.1038/nn1228 15114358

[B2] Stevenson IH Kording KP How advances in neural recording affect data analysis Nat Neurosci 2011 14 139 142 10.1038/nn.2731 21270781 PMC3410539

[B3] Buzsáki G Large-scale recording of neuronal ensembles Nat Neurosci 2004 7 446 451 10.1038/nn1233 15114356

[B4] Denker M Wiebelt B Fliegner D Diesmann M Morrison A Practically trivial parallel data processing in a neuroscience laboratory Analysis of parallel spike trains 2010 New York: Springer-Verlag

[B5] CARMEN Code analysis, repository & modeling for e-neuroscience http://www.carmen.org.uk

[B6] Herz AVM Meier R Nawrot MP Schiegel W Zito T G-Node: An integrated tool-sharing platform to support cellular and systems neurophysiology in the age of global neuroinformatics Neural Networks 2008 21 1070 1075 10.1016/j.neunet.2008.05.011 18653312

[B7] CRCNS Collaborative research in computational neuroscience http://crcns.org/

[B8] Nordlie E Gewaltig MO Plesser HE Towards reproducible descriptions of neuronal network models PLoS Comput Biol 2009 5 e1000456 19662159 10.1371/journal.pcbi.1000456 PMC2713426

[B9] Sumatra: automated electronic lab book http://neuralensemble.org/trac/sumatra

[B10] LONI Pipeline http://pipeline.loni.ucla.edu/

[B11] VisTrails [http://www.vistrails.org/]; Taverna [http://www.taverna.org.uk/]; Kepler [https://kepler-project.org/]

